# Dr. Henry Drury Noyes

**Published:** 1900-12

**Authors:** 


Dr. Henry Drury Noyes, of New York, died of pneumonia at Mount Washington, Mass, November 12, 1900, aged 68 years. He was famous as a teacher and author and was among the distinguished ophthalmologists of the period. He was active in his work as practitioner, teacher and writer until his last illness.

